# A framework for a low‐cost system of automated gate control in assays of spatial cognition in fishes

**DOI:** 10.1111/jfb.15958

**Published:** 2024-10-17

**Authors:** Valerie Lucks, Jens Theine, Maria Paula Arteaga Avendaño, Jacob Engelmann

**Affiliations:** ^1^ Active Sensing, Faculty of Biology Bielefeld University Bielefeld Germany; ^2^ Genetics and Genomics of Plants, Faculty of Biology & Center for Biotechnology Bielefeld University Bielefeld Germany

**Keywords:** automation, motor system, navigation, raspberry pi, setup gates, spatial learning, weakly electric fish

## Abstract

Automation of experimental setups is a promising direction in behavioral research because it can facilitate the acquisition of data while increasing its repeatability and reliability. For example, research in spatial cognition can benefit from automated control by systematic manipulation of sensory cues and more efficient execution of training procedures. However, commercial solutions are often costly, restricted to specific platforms, and mainly focused on the automation of data acquisition, stimulus presentation, and reward delivery. Animal welfare considerations as well as experimental demands may require automating the access of an animal or animals to the experimental arena. Here, we provide and test a low‐cost, versatile Raspberry Pi‐based solution for such use cases. We provide four application scenarios of varying complexities, based on our research of spatial orientation and navigation in weakly electric fish, with step‐by‐step protocols for the control of gates in the experimental setups. This easy‐to‐implement, platform‐independent approach can be adapted to various experimental needs, including closed‐loop as well as field experiments. As such, it can contribute to the optimization and standardization of experiments in a variety of species, thereby enhancing the comparability of data.

## 1 INTRODUCTION

Studying cognitive functions and learning in animals can be time‐consuming and labor‐intensive. Automation may be advantageous in such studies as it can shorten the time needed for training and data acquisition (Buatois et al., 2024). In addition, automation can reduce unintended cueing by the experimenter. Requiring standardized conditions, it can further help in making results more reproducible and comparable across laboratories and species (Würbel, 2000). Arguably, automation will be most beneficial where high‐throughput behavioral screening is required (e.g., Creton, 2009; ManyFishes project, see Nelson, 2024; Li, Hao, et al., 2022; Li, Wu, et al., 2022; Ramborger et al., 2024) or when an established learning protocol is repeatably being used (e.g., Behrend et al., 1968). Despite the obvious potential benefits of automation, manually operated and often large setups remain the standard in fish research (Ajuwon et al., 2024). In part, this can be attributed to the considerable technical expertise and effort required for automation. However, driven by the surge in zebrafish research (Teame et al., 2019), there are now excellent tools that allow fully automated tracking and closed‐loop experiments in fish (Deakin et al., 2019; Guilbeault et al., 2021; Ajuwon et al., 2024; Stowers et al., 2017; Parker et al., 2013).

Automation has been successfully applied across various fish species and learning paradigms (Barreiros et al., 2021; Behrend et al., 1968; McKay et al., 2022; Pylatiuk et al., 2019; Singh et al., 2022). A recent study (Buatois et al., 2024) found a significant improvement in automated associative learning of zebrafish compared to the classical manual protocol (Colwill et al., 2005). We are only aware of two studies that directly compared the performance of fish in automated and manual training (Gatto et al., 2020; Gatto et al., 2021) and these reported poorer learning performance for some of the automated tasks. However, there were significant technical differences in the apparatuses used for the automated and manual training which, as argued by the authors, could have resulted in a higher “cognitive load” in the automated version.

Behavioral experiments should align with the sensory and cognitive abilities of the species under investigation. When data on these factors is limited, as in the case of spatial learning in weakly electric fish, training protocols may need to be adapted based on the experimenters' experience with the species and paradigm. This led us to develop a stepwise approach transitioning from manual to automated training. We present a modular, versatile, and inexpensive approach that allows varying degrees of automation in controlling the fish's access to experimental arenas, a common requirement in fish studies.

This approach is motivated by our studies on spatial navigation of the weakly electric black ghost knifefish, *Apteronotus albifrons*. These gymnotiform fish live in shallow freshwater rivers of the Neotropics and are known to leave their shelters at nightfall for long foraging excursions (Hagedorn, 1988; Hopkins, 1974; Raab et al., 2019; Steinbach, 1970; Westby, 1988). Exactly how they navigate the rivers in darkness is still not fully understood. Amongst other senses common to all teleosts, these so‐called weakly electric fish continuously discharge an electric organ to create an electric field surrounding their body (Bastian, 1982; Nelson & MacIver, 1999). The small perturbations caused by nearby objects in the fish's electric field are sensed by specific cutaneous electroreceptors that provide information about an object's location, size, and electrical properties (von der Emde et al., 1998). While this highly specialized sense only operates in the near range of the animal, other senses like olfaction, hydrodynamic perception, and vision may be used in navigation (MacIver et al., 2001). With a resolution of 0.57 to 0.54 cycles per degree (cpd, Takiyama et al., 2015) and dim‐light adapted visual pigments (rhodopsin, Van Nynatten et al., 2019), *Apteronotus albifrons*' visual system is optimized for its nocturnal lifestyle and might therefore be supplementing the near‐range electrosensory information with far‐range information (MacIver et al., 2001). We aimed to understand which senses contribute to navigation in weakly electric fish, specifically in black ghost knifefish. Investigating whether these fish can estimate swim distances requires a large arena, and controlling rewards and access can be challenging for a single experimenter. Furthermore, any interaction with the experimenter can create unwanted cues, whether by moving around the tank to place food rewards, standing at a position that might act as a landmark, or unintentionally revealing correct choices (Samhita & Gross, 2013), all of which may influence the animal's behavior (Chakravarty et al., 2013; Conde‐Sieira et al., 2018; Egan et al., 2009; Metcalfe et al., 1987; Ramsay et al., 2009). Our experiment therefore benefits from versatile automation as it allows manipulation and control of different parts of the experimental arena from a distance.

Similar to other studies in fish (Gatto et al., 2020; Gatto et al., 2021; Martorell‐Barceló et al., 2021; Randlett, 2023; Tomihara et al., 2021), we use the low‐cost single‐board computer Raspberry Pi (Raspberry Zero W) to control electronic components of the setup. It is well suited for researchers with different levels of programming experience and enables users to implement non‐proprietary user‐defined functionality through Python. Its general‐purpose input/output pins (GPIOs) allow for the connection of various hardware, in our case, motors and pushbuttons. In contrast to simple microcontrollers, the Raspberry Zero W possesses a wireless connectivity module (Upton & Halfacree, 2014; Upton & Halfacree, 2016), which we used to create a local hotspot to operate the system remotely. This is a valuable addition to previous designs (Aoki et al., 2015; Buatois et al., 2024; Doyle et al., 2017; Jung et al., 2019) because it allows for an independent, remote communication with the system, making it also suitable for fieldwork (Häderer & Michiels, 2024; Henninger et al., 2018).

**FIGURE 1:**
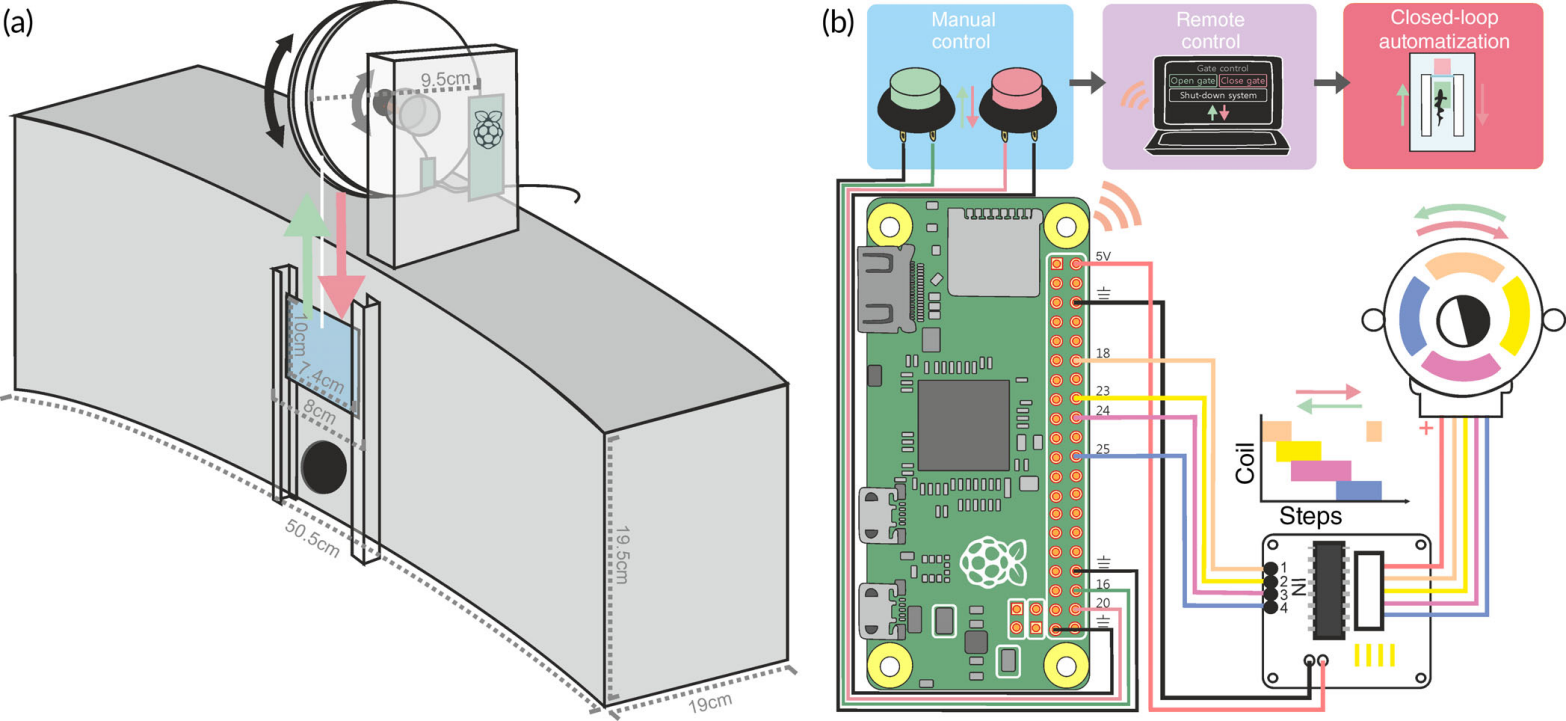
Mechanics of the setup gate system. (a) Gate system consisting of a Raspberry Pi Zero W and a stepper motor connected to a spindle disk to lift or lower the plastic partition in front of the entrance hole in the home compartment. This system was placed on top of the fish's home compartment and used to regulate when fish could access the experimental arena. (b) The modular design of the system allows for gate control of increasing complexity: manually via pushbuttons, remotely via a web interface on a hotspot created by the Raspberry Pi, as well as in closed‐loop mode when coupled to video acquisition software checking for the presence of the fish in regions of interest (see colored boxes on top). The wiring scheme indicates GPIO pushbuttons and motor connections as used, and further detailed, in the code provided in the Supporting Information (Appendices 1‐5). The motor, powered and driven by the motor controller, rotates as GPIO output activates the four coils sequentially (see inset). Reversing the sequence order reverses the motor's rotation.

This paper presents four applications featuring increasing levels of automation, ranging from simple pushbutton control to remote control and closed‐loop procedures (Figure 1). We provide preliminary data on distance learning in *Apteronotus albifrons* and assess the possible effects of the automated gates on the fish. Detailed step‐by‐step instructions and all code are included, enabling users to replicate our approach and customize it to meet their individual needs.

## 2 METHODS

### 2.1 Training procedure

The experiments were divided into two phases: a training phase and an experimental phase. The training phase used the simplest version of the automated gate system (pushbutton control), whereas the test phase required remote observation and control of the gate (Figure 1).

#### 2.1.1 Training phase

**FIGURE 2:**
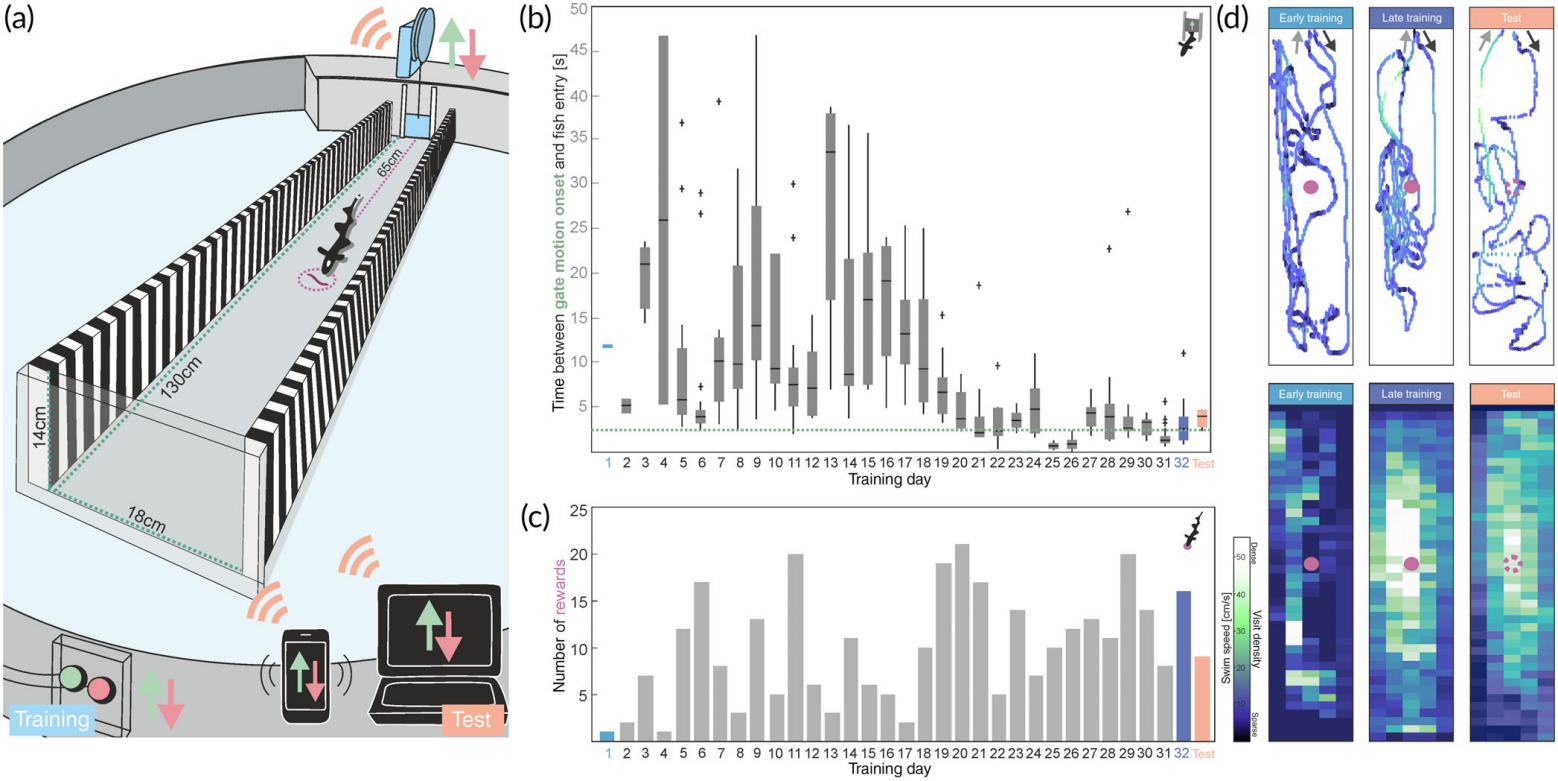
Distance estimation learning. (a) Schematic of the experimental arena. The swim tunnel was positioned within a circular tank (180 cm diameter). While a featureless black cloth surrounded the walls and the ceiling of the experimental room to prevent external visual cues, the walls of the swim tunnel showed a vertical stripe pattern (bars of 2 cm width). The home compartment which housed the fish between trials was completely covered by a PVC plate and connected to the swim tunnel by a non‐transparent automated gate to prevent the fish from seeing when and where the food reward (single mosquito larva) was placed in the tunnel. The reward was always placed at a distance of 65 cm from the gate in the middle of the swim tunnel. Gate motion was controlled by a Raspberry Pi located above the home compartment. Gate opening and closing could be triggered either locally through the pushbuttons attached to the opposing end of the swim tunnel, or remotely by use of WIFI‐capable devices. (b) Gate characteristics and their effect on fish behavior throughout the training phase. The distance, speed, and duration (set here to 2.4 s) of gate movement are defined in the code and depend on the actual dimensions of the pulley system. The accuracy of the gate's timing was verified by tracking the motor's indication LED in training and test video data (green dashed line). The delay between the onset of the opening of the gate and the time the fish entered the swim tunnel decreased with training (grey boxplots, linear regression slope <0, *F* = 15.7, *p* < 0.05), reflecting the habituation of the animal to the operation of the gate and the successful training. (c) Performance of the fish throughout training. With training days, the fish completed more successful trials (i.e. leaving the home compartment, locating and feeding on the reward, and returning to the home compartment; linear regression slope >0, *F* = 12.2, *p* < 0.05) within the 20‐min training period. This performance persisted in unrewarded test trials. (d) Example of the fish's behavior in early and late training and test trials. Fish focused their spatial attention on the food reward in early and late training as well as in unrewarded test trials. This can be seen both in the representative single trajectories with the swim speed being color‐coded (top) and in the heatmaps pooling all trials of the respective sessions (center). (Note the indication of sessions [training day 1, 32, and ‘test’] used as examples for early training, late training, and tests in (b) and (c).) There was no change in swim speed when leaving the gate (outbound, indicated by dark grey arrows) nor around the target, but a slight increase in inbound (indicated by light grey arrows) movements in late training. (b‐d) Data from a single representative fish.

Fish were trained to localize a food reward (single mosquito larva) within a 130‐cm long swim tunnel (Figure 2a). The tunnel walls showed a vertical stripe pattern to investigate the contribution of sensory flow (optic or electric) to distance estimation. The food reward was presented at a fixed distance of 65 cm from a home compartment that housed the fish between experimental trials. Access to the swim tunnel was regulated through the automated gate. Once the gate was opened, the fish could enter the swim tunnel and explore it freely. When the fish returned to the home compartment the gate was closed, the reward was replaced, and a new training trial started. This was repeated for 20 min to keep the fish motivated by food and avoid overtraining. A training session was aborted when the fish did not return to the home compartment within 20 min. This was only observed during the early training phase in one individual. At the end of a session, fish were moved back to their home tank without netting, i.e., they swam in a transport tank placed within the home compartment to ensure non‐harmful transport of the fish.

In this training phase, the gate was controlled via the Raspberry Pi‐based system using simple pushbuttons (Figure 1, and Videos S1 and S2). Detailed instructions including Python code are provided in the Supporting Information (Appendices 1‐3). These buttons allowed the experimenter to remain far from the home compartment, minimizing stress for the fish while still allowing direct behavioral observation. We expected that this, together with the consistent movement of the automated gate, should facilitate task acquisition as black ghost knifefish are known to be difficult to train (Dangelmeyer et al., 2016). To quantify how fish responded to the automated opening of the gate, we analyzed (i) the time the fish took from the onset of gate motion until it was detected inside the swim tunnel, (ii) the consistency of the gate's movement as the time it took to open the gate completely in each training trial, (iii) the speed at which the fish entered their home compartment during both the inbound and outbound trajectories, and (iv) the probability of visually observable freezing behaviors of the fish, a response typically considered to be stress‐related (Houslay et al., 2022; Kalueff et al., 2013).

The gate was built from a non‐buoyant lightweight PVC plate (1.2 g cm^−3^, 2.8 × 10 × 0.3 cm, 26.6 g). This gate could be opened and closed with a motorized pulley system (Figures 1 and 2a, Figure S1), positioned so as not to interfere with post hoc tracking of the fish's position.

#### 2.1.2 Testing phase

Once fish were sufficiently acquainted with the task, we tested their ability to target the previously food‐rewarded location. These tests were conducted in a separate identical swim tunnel that never contained any food reward to avoid potential chemical cues. Additionally, we tested for the contribution of other external sensory cues by rotating the swim tunnel (test for allocentric visual cues) and conducting the test in a separate tank in a neighboring room (test for non‐visual allocentric cues). Additionally, all visual and electric cues (i.e., the stripes on the swim tunnel walls) could be removed to test for the contribution of optic and electric sensory flow. All tests required the experimenter's absence from the experimental arena. This required us to observe the fish remotely and control the gate accordingly. Video‐based control of the fish's behavior was achieved through Matlab's video input (VT Marlin F033B, Stemmer Imaging, 1024 × 768 pixels, 30fps). While the pushbutton control in principle could also be used in such a scenario, we opted for a more flexible wireless solution: by creating a WIFI connection through a hotspot on the Raspberry Pi, the gate can be controlled with any WIFI‐capable device independently of a laboratory's layout and equipment, in principle even in field research. Specifically, the Raspberry Pi was set up as a local web server providing a Hypertext Preprocessor (PHP)‐based graphical user interface (GUI). Virtual button presses in this GUI trigger the execution of Python scripts on the Raspberry Pi (Figure 1) that open/close the gate. Appendix 4 in the Supporting Information provides detailed instructions to implement this approach, including all code.

### 2.2 Ethics statement

Fish were kept in groups in 350‐L tanks under a 12/12 h light/dark cycle. The tanks were equipped with shelters and plants. The water in both the experimental and housing tanks was kept at a conductivity of 120 ± 5 μS/cm and a temperature of 22 ± 2°C, and was regularly checked for chemical and physical parameters.

All procedures for animal maintenance and experimentation complied with the current animal protection law of the Federal Republic of Germany, approved by the local authorities Landesamt für Natur, Umwelt und Verbraucherschutz Nordrhein‐Westfalen (approvals 84‐02.04.2017.A151 and 87‐51‐04.2010.A202).

## 3 RESULTS

The near‐range nature of the electric sense and the resulting tight link between motor behavior and sensing in weakly electric fishes makes them interesting subjects for the study of spatial navigation (Engelmann et al., 2021; Fotowat et al., 2019; Jun et al., 2016; Wallach et al., 2018). We studied if and how the weakly electric gymnotiform fish *Apteronotus albifrons* measure distance, a central ability for spatial orientation that was only recently documented to exist in fish (Karlsson et al., 2022; Sibeaux et al., 2022). In the following, we present data demonstrating the suitability of the automated gate system in training and testing the spatial learning of *Apteronotus albifrons*.

Fish took less time to enter the arena after gate opening with prolonged training, indicating that they learned to interact with the automated gate (Figure 2b). The performance (i.e., the number of trials in which a fish located the rewarded position per session) improved parallel to the reduced latency to enter the tunnel, as indicated by the increased number of larvae consumed per experimental session (Figure 2c). In this representative fish, both parameters reached a stable level after 18 training days. For the five fish tested, this took between 19 and 27 days after which the performance remained stable, also in the unrewarded test trials (Figure 2b,c). Occasionally fish entered the arena even before the gate had opened completely (e.g. Figure 2b, day 25, gate motion took 2.4 ± 0.05 s), which further indicates that the gate itself was not perceived as harmful. The swim speed of the fish within the swim tunnel was indistinguishable from that when fish entered or left the tunnel (Figure 2d) but slightly increased during inbound trajectories of the late learning phase and test trials. In no case did any fish exhibit freezing, a fear‐ and stress‐related behavior (Jesuthasan, 2011), near the gate. As shown by the visit density maps for the first and last session of the learning phase, fish also learned to localize the food reward more accurately (Figure 2d).

A total of five fish were trained and then tested for the ability to measure the distance to the target (see exemplary data in Figure 2d). These tests made use of the WIFI‐controlled gate, which showed a delay between the virtual pushbutton press and the onset of the gate opening of 0.6 ± 0.002 s. In no cases did we encounter any malfunction of the system.

### 3.1 Perspective: fully automated condition

**FIGURE 3:**
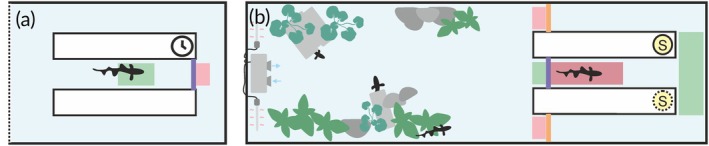
Illustration of potential applications of the automated motor system. A hotspot‐based connection to the Raspberry Pi enables remote gate control, thereby facilitating the complete automation of the experiment and data acquisition. (a) Top view of an experimental arena with a single gate ( blue ) and two ROIs (green and red, respectively). The video‐based automated detection of the fish in either ROI triggers both the video acquisition and opening (green ROI) or closing (red ROI) of the gate. This simple setup enables randomization of the interval between entering the ROI and gate opening to investigate the short‐term memory capacity of these fish. Similarly, this approach could be modified to regulate the timing or type of stimuli being presented (the methodology for this approach is outlined in the Supporting Information, Appendix 5). (b) Expansion of the approach to a T‐maze with three gates ( blue and orange) and five ROIs. As indicated by the schematic of multiple fishes in the home‐tank region of the setup, this approach could be used in communal “home‐tank” experimental paradigms, obviating the need to isolate individuals. Here the entrance gate ( blue ) controls the access of a fish to the maze where stimuli are presented and also starts the video acquisition. On entering, the individual can be identified based on visual features (e.g., size, markers, feature extraction by deep learning algorithms, red ROI). Once a fish enters the top junction ROI of the maze the two exit gates (orange) are opened. The fish, trained to either swim left or right in response to a stimulus (S), makes its decision by entering one arm, which then triggers another ROI (red) leading to the closing of the exit gates and the end of the video acquisition (see Figure S2, further instructions for this approach are provided in the Supporting Information, Appendix 6).

Next, to establish a closed‐loop approach, we demonstrated an incremental increase in the automation of the gate. The experiment was designed to investigate the working memory span in weakly electric fish (no data shown). In this setup, fish can be trained to pass through a T‐maze‐like setup where the access to the two decision arms is blocked by a gate. When approaching this gate, the fish passes stimuli (e.g., electrically prominent objects) that are positioned alongside the tunnel and enters into a first region of interest (ROI) (Figure 3a, green area), which was continuously monitored for the fish's presence. Detection of a fish in this ROI triggered the video acquisition (delay of 0.27 ± 0.005 s after fish detection [afd]) and the opening of the gate (delay of 0.3 ± 0.005 s afd). Note that this time includes the initialization of video acquisition and is, therefore, longer than any subsequent gate activation, and that all delays reported here depend on the performance and settings of the hardware (camera and PC) and not on the Raspberry Pi's gate control software. By adding a user‐coded delay to the opening of the gate, it was possible to investigate memory‐related parameters, i.e. the duration over that fish can recall on which side of the tunnel an object was located. Fish can be trained to indicate this side by turning either left or right at the end of the tunnel, where a food reward may be placed in the training phase. Once the fish passes the gate, this triggers a second ROI that stops the video acquisition and initiates the gate's closing (delay of 0.0032 ± 0.0005 s afd) (Figure 3a; for detailed instructions and code, see Appendix 5 in the Supporting Information).

The modularity of our approach is illustrated in the following, in which we extended our approach to operate in a closed‐loop setting. (For detailed instructions and code, please refer to Appendix 6 in the Supporting Information, Figure 3b, and Figure S2). In this configuration, fish were permitted to access the T‐maze via an entrance gate controlled by two regions of interest. One of these ROIs initiated the video acquisition with a delay of 0.28 ± 0.005 s afd and the opening of the gate. The second ROI was responsible for initiating the closing of the gate, which occurred with a delay of 0.0035 ± 0.0005 s afd. Again, stimuli could be presented on either side of the swim tunnel. Once the fish reached the third ROI at the end of the T‐maze it made a decision by either swimming to the left or right arm of the maze, which triggered the opening of both exit gates (delay of 0.0036 ± 0.0005 s afd). It is important to note that achieving such synchronous timing of two gates was a challenging task when attempted manually. On the fish's passage through either of the exit gates, they closed (delay of 0.0035 ± 0.0005 s afd), and video acquisition was stopped and restarted once the fish re‐entered the entrance gates (Figure 3b). The closed‐loop design of the setup renders it particularly well suited for “in‐tank” experimental designs, including communal housing of fish (see Discussion, Figure 3b).

In this study, we did not implement automated reward delivery as our fish were fed and rewarded with semiliquid food (red mosquito larvae) that cannot easily be dispensed with motorized feeders used in other studies (Ajuwon et al., 2024). Where required, however, automated custom‐built peristaltic feeders could be included in the design of the experiment (Mueller & Neuhauss, 2012).

## 4 DISCUSSION

Tests addressing the cognitive capabilities of fish are frequently conducted in specialized setups that, similar to rodent research, require the transfer of animals that may result in aversive handling effects (Brydges et al., 2009). Many experiments also require extensive intervention by the experimenter during training and data acquisition, making them susceptible to what has been referred to as “experimenter effects” (Bohlen et al., 2014; Lang et al., 2023; Samhita & Gross, 2013). In fish research, the experimenter is often required to stay in visual proximity to the fish for behavioral observation, to move around the setup to operate gates, position food rewards, or even directly reward the fish in an experiment. Besides potentially constituting a landmark for fish in experiments focusing on spatial learning, experimenter interactions may also induce fear responses, adversely interfering with the goals of a study (Buatois et al., 2024; Hulthen et al., 2021).

Automation can help overcome the experimenter effect and has been implemented in data acquisition and reward delivery in training paradigms for a variety of animals (Choi, 2018; Steurer et al., 2012), including fish (Barreiros et al., 2021; McKay et al., 2022; Pylatiuk et al., 2019; Singh et al., 2022). Not surprisingly, however, there are only a few studies aiming to directly compare manual and automated conditioning in fish. Notably, a recent study in guppies (Gatto et al., 2021) reported poorer performance in automated versus manually conducted tests while another study in zebrafish (Gatto et al., 2020) found that the saliency of stimuli used in automated visual experiments may be a key factor in determining the learning success. Both studies demonstrate that the experimental design should be matched to the species being investigated. While we are not aware of studies addressing this in fish directly, Prétôt et al. (2016) showed that the poor performance of primates in a two‐choice discrimination task that was easy for fish (Salwiczek et al., 2012) could be explained by the task not being adapted to species‐specific strengths (ecology and motor repertoire) (Prétôt et al., 2016).

Even within a species, persistent individual behavioral differences are found, and learning can be affected by such interindividual differences (Conrad et al., 2011; Nomakuchi et al., 2009; Sneddon, 2003). Not only should the experimental design be matched to a species' ethology, but it may also benefit from adjusting a paradigm to individuals. Accounting for such subtle differences with a fully automated approach may be difficult, especially when working with non‐model fish species where we know less about their ecology and behavior. Arguably it is the strength of the human experimenter to gauge the individual's needs to optimally learn a novel task. Once ideal training parameters have been established by an experimenter, or when individuals have reached a stable performance level, automating the actual tests can help to reduce experimenter effects and make tests and therefore the results more reproducible.

The objective of our approach was the development of a gate system that could be rapidly adapted to different requirements. This included the creation of a human‐experimenter‐controlled version for training purposes, a remotely operated version for testing, and a closed‐loop control scenario. Compared to the time required for manual training of weakly electric fish (Dangelmeyer et al., 2016), no difference was observed in the time it took the five fish tested to reach a stable performance in the automated gate setup (Figure 2b,c). This, together with the absence of anxiety‐related freezing behavior (Jesuthasan, 2011), suggests that the fish readily adapted to the motorized gate despite the motor system being situated directly above the setup. Experiments on more sensitive species that require a better isolation from vibrations may benefit from placing the stepper motor further from the setup, which can be achieved by elongating the pulley system.

To ensure the animals' welfare, the gate was constructed from a lightweight PVC plate (26.6 g) with a density only slightly greater than that of water (1.2 g cm^−3^). The slow opening and closing speed, as defined by the code (2.9 cm s^−1^), ensured that minimal forces would be exerted on the fish in the event of contact with the moving gate. Additionally, the low weight of the gate prevented the fish from becoming trapped by the gate. When working with smaller species, it may be advisable to incorporate sensory feedback from optic or load sensors to include some form of touch‐specific interrupt. At present, a basic security mechanism is in place that compares the number of completed steps during opening and closing (as well as distinct prompt states for “open” and “close”, shown in the Supporting Information, Appendix 6) to prevent the accumulation of missed steps or false triggers that could result in a malfunction of the gate. As shown, this proved effective in our case.

The ability to control the gate locally through wired pushbuttons or remotely using WIFI connectivity proved to be advantageous in the gradual transition between the learning and test phases in our experiments. As demonstrated in the closed‐loop condition, the system operates with negligible lag and thus can also be used in more challenging conditioning scenarios that require precise timing between the behavior of the animal and the operation of the gate(s). It thus can be included as a crucial part of “home‐tank” experimental designs to regulate access to the experimental part of a tank. The frequently employed method of individually examining animals in a test apparatus outside their home cage or tank can influence the experimental outcome as handling, the new arena, social isolation, and the time point of the experiment may all impact the behavior or motivation of an animal to participate. Increasingly, research thus proposes “home cage‐based” experimental paradigms (Buatois et al., 2024; Doyle et al., 2017; Kahnau et al., 2023). In fish research, such “home‐tank” approaches are more commonly used, probably due to the need to keep individuals separate without tagging. A positive side‐effect of these home‐tank approaches is that they avoid the daily and potentially stressful handling and transport of the fish by the experimenter (Pavlidis et al., 2013). While social isolation in these approaches may negatively affect the learning performance in some fish (Lombardi Brandão et al., 2015), stress levels were found to be lower in isolated zebrafish compared to communal tanks (Parker et al., 2012), suggesting that species‐specific home‐tank conditions should be established prior to the design of an experiment.

Adding a closed‐loop version of the gate to home‐tank‐based fish conditioning setups (e.g., Ajuwon et al., 2024; Buatois et al., 2024; Doyle et al., 2017) has the potential to enhance the efficacy of methods in fish research (Figure 3b). As an example, the integration of the gate system into a home‐tank compartment (see Supporting Information, Appendix 6) may facilitate the automated commencement of training and data acquisition, thereby ensuring that the training and data acquisition processes are regulated by the motivation of the individual to engage in the experiment. Furthermore, the integration of a visual system for individual discrimination within a communal tank could potentially reduce the necessity for animal isolation during such experiments (Figure 3b). For smaller fish this is challenging, but recent advances in makerless pose estimation through extended deep learning algorithms (e.g., DeepLabCut environment) offer a means of extracting relevant features to discriminate individuals (Lauer et al., 2022; Mathis et al., 2018). Where animal welfare considerations permit, RFID tagging may also be implemented with either single‐board computers (Amora et al., 2020) or microcontrollers (Bridge et al., 2019).

Automated systems can be fairly sophisticated (Dutta et al., 2023; Mueller & Neuhauss, 2012; Parker et al., 2013; Stowers et al., 2017) but rely on advanced programming skills that limit their distribution in research communities. The approach described here prioritizes the use of low‐cost components, flexibility to align with the specific requirements of individual laboratories, and complete accessibility of the code required. It should be noted that we do not propose a standalone approach to automation in fish research. However, our gate system can be readily integrated into existing laboratory routines, particularly when automation is developed concurrently with the experimental progress. For researchers newly establishing training procedures with a need to integrate data seamlessly from various input and output streams (camera, microcontrollers, stimuli), the visual programming environment Bonsai (https://bonsai-rx.org/) may be an appropriate option. Its suitability for closed‐loop experimentation in fish research was recently demonstrated (Ajuwon et al., 2024), and our gate system could be incorporated and controlled with ease in this environment. The protocols to implement the different paradigms presented in this work are provided in the Supporting Information (https://github.com/vlucks/Automated_Setup_Gates) and should facilitate an easy and cost‐effective expansion of tasks. This includes the addition of further sensors to acquire environmental parameters (Sheikh & Xinrong, 2014) or the expansion of the interactive scope of the experiments (Givon et al., 2022) to include touch sensors for operant conditioning (Ardesch et al., 2017; Buscher et al., 2020; Moro et al., 2023; O'Leary et al., 2018; Silasi et al., 2018) or visual displays (Gurley, 2019). The Raspberry Pi Zero W used here was chosen for its optimal cost–benefit ratio and the built‐in WIFI chip. For more demanding projects, more powerful Raspberry Pi models (e.g., Raspberry Pi 5, 64‐bit quad‐core, 2.4 GHz processor, and a graphics performance of 800 MHz; Raspberry Pi, 2023a) might be a good choice. Such a system then could also be coupled to a dedicated camera module (e.g., Raspberry Pi 12MP HQ camera with Sony IMX477R Sensor; Raspberry Pi Ltd, 2023) for onboard video and data acquisition. Examples of use cases integrating video input include work in insects (Cano‐Ferrer et al., 2023; Krupa et al., 2021; Tadres et al., 2024a, 2024b; Tadres & Louis, 2020), amphibians (Groffen & Hoskin, 2024), rodents (Centanni & Smith, 2023, Vassilev et al., 2020; Benedict & Cudmore, 2023), birds (Hereward et al., 2021), and fish (Signaroli et al., 2022; Todd et al., 2017; Li, Hao, et al., 2022; Li, Wu, et al., 2022; Randlett, 2023; Martorell‐Barceló et al., 2021). Following the WIFI remote control documented in this study, such stand‐alone systems could be an excellent choice for field work of neurobiological approaches (e.g., Campbell & Jones, 2020; Maia Chagas et al., 2017).

Taken together, we reported on the integration of a versatile and low‐cost gate system to regulate the entry of fish into an experimental arena. The management of this gate can be automated gradually, allowing us to train and test spatial learning in weakly electric fish with reduced human interference and adaptability to individual needs during different phases of an experiment. The system's modular design allows for customized solutions addressing various experimental approaches with different levels of automation.

## AUTHOR CONTRIBUTIONS

V.L. and J.E. designed the experiment. V.L. and J.T. implemented the system. V.L. and M.P.A.A. tested the system. V.L. and J.E. wrote the paper.

## FUNDING INFORMATION

This research was partially funded by a Human Frontier Science Program grant RGP0016/2019.

## Supporting information


**S1.** Supporting Information.

**VIDEO S1.** Top view recording of a test trial showing how a representative *Apteronotus albifrons* enters the swim tunnel through the automated gate (the time of gate opening is indicated by the LED), searches for a reward at the trained location (asterisk), and re‐enters the home compartment.

**VIDEO S2.** Underwater recording of a training trial showing how an individual *Apteronotus albifrons* enters the swim tunnel shortly after the automated gate opens to feed on the previously positioned reward.
