# Supplementary material for: A framework for a low‐cost system of automated gate control in assays of spatial cognition in fishes
Source: J Fish Biol. 2024 Oct 17;107(4):1129–38. doi: 10.1111/jfb.15958 (PMC12536059; doi:10.1111/jfb.15958)
Supplement: Supplementary file 1 — S1. Supporting Information. [file JFB-107-1129-s002.docx]

Supporting Information:

A framework for a low-cost system of automated gate control in assays of spatial cognition in fishes

**Appendix 1: Headless initialization of a Raspberry Pi**

Required parts:

1. Raspberry Pi Zero W single-board computer
2. 3A Micro USB power supply (5.1V DC, 3.0A)
3. 32 GB Micro Secure Digital (SD) card

Protocol:

On your PC:

1. Insert the microSD card into your PC’s SD card adapter.
2. Download the latest Raspberry Pi image “Raspberry Pi OS (32-bit) Lite” from https://www.raspberrypi.org/downloads/raspberry-pi-os/.
3. Expand the downloaded zip file (e.g. using the program 7zip, https://7-zip.de/download.html).
4. Install Win32DiskImager (https://win32diskimager.org) to write the image to the SD card.
   1. Open Win23DiskImager.
   2. Select the downloaded, unpacked Raspberry Pi image and choose the SD card as ‘Device’ (Be careful not to select /C or any other drive not to overwrite system files).
   3. Write the image to the SD card.
5. To modify the Raspberry Pi wirelessly, it needs access to your home wifi network. To do so, install the program WinSCP (https://winscp.net/eng/downloads.php).
   Access to your home network is defined in two created files:
   1. Create one file named ‘ssh’ without any content (make sure not to specify any file extension).
   2. The second file named ‘wpa_supplicant’ should contain all network access information to the home wifi. Customize the 2-digit country code, ‘Name of Network’, and ‘Password’ to your network settings.

country=DE # Your 2-digit country code
ctrl_interface=DIR=/var/run/wpa_supplicant GROUP=netdev
network={
 ssid="NameOfNetwork"
 psk="Password”
 key_mgmt=WPA-PSK
}

- 1. Drag and drop both files into the SD card directory using WinSCP.

1. Now your SD card is ready to go – safely remove it, insert it into the Raspberry Pi’s SD card slot, and connect the power supply to the Pi. When you see the Raspberry Pi’s green LEDs flashing, the operating system is booting.
2. Check which IP address is assigned to the Raspberry Pi. The easiest way is to check the clients list in your network (e.g. by typing ‘http://fritz.box/’ in any browser).
3. Install putty (https://www.putty.org/).
   1. Enter your Raspberry Pi’s IP address as a hostname and open the connection.

On the Raspberry Pi’s terminal:

1. A terminal is going to open with which you can shell-code directly on the Raspberry Pi.
   1. By default, the user is ‘pi’ and the password is ‘raspberry’
2. To finalize the initialization, crucial security settings must be set. Execute
   ‘sudo raspi-config’ to open an interface to customize the Pi’s username and password; also define your location.
   1. Execute ‘sudo apt update’ & ‘sudo apt upgrade’ to get all available updates for the Pi.
   2. Execute ‘sudo apt install fail2ban ufw.
      1. Fail2ban is exponentially increasing waiting time between false password attempts.
      2. Carry out ‘sudo ufw allow 22/tcp’ to communicate with the Pi via port 22.
      3. In case you are using this protocol to continue the remote-controlled version of the gates (Appendix 4-6), execute ‘sudo ufw allow 80/tcp’ to permit web server communication. To activate the firewall, execute
         ‘sudo ufw enable’.
3. Shut down the Raspberry Pi by executing ‘sudo poweroff’.

**Appendix 2: Motor connection and gate mechanics**

In addition to the equipment listed in Appendix 1, the following parts are used:

1. GPIO header
2. GPIO cables
3. Stepper motor 28BYJ-48 with ULN2003 motor driver
4. PVC plate as the gate’s door
5. Fishing string to attach the gate to the motor
6. PVC U-profile as guide rails for the gate
7. Spindle disk (custom-made or 3D printed)
8. Soldering iron

Protocol:

1. Perform all steps mentioned in Appendix 1.
2. Solder the GPIO header to the Raspberry Pi’s GPIOs.
3. Connect the stepper motor to the motor driver.
4. The motor is driven by sequential activation of magnetic coils (Figure 1B):
   1. Accordingly, all 4 motor driver’s pins need to be connected to 4 free GPIOs (any GPIO can be used except for ground and power supply, see Figure 1B) on the Raspberry Pi. For example, connect the motor driver’s IN1 to Pin12 (GPIO18), IN2 to Pin16 (GPIO23), IN3 to Pin18 (GPIO24) and IN4 to Pin22 (GPIO25). Figure 1B provides a wiring scheme. Remember which GPIOs you used as those need to be defined in the Python code.
   2. To supply the motor driver with power, connect the ‘+’ pin of the driver board to a 5V power supply (i.e. pin2) and the ‘-‘ pin to ground (i.e. Pin6) (Figure 1B).
5. Resume editing the software by plugging the Raspberry Pi back into the power supply, wait for it to boot and connect to the Raspberry Pi OS via ssh using putty (see Appendix 1, step 8).

On the Raspberry Pi’s terminal:

1. Execute ‘nano motor_open.py’ to create a Python script.
   1. Copy the ‘motor opening’ code provided at https://github.com/vlucks/Automated_Setup_Gates/blob/main/Appendix2_MotorConnectionandGateMechanics/Appendix2_motor_open.py
      (modified after Joy-it, 2017).
   2. Save and close the file. Adapt the GPIO indexes in case you deviated from the examples mentioned above; pay attention to follow the order given by the motor driver's pins IN1-4.
2. Execute ‘nano motor_close.py’ to create ‘motor closing’ python script.
   1. Copy the same code as above but reverse the sequence of steps to rotate the motor in the opposite direction, then save and close the script. Alternatively, use the code provided at https://github.com/vlucks/Automated_Setup_Gates/blob/main/Appendix2_MotorConnectionandGateMechanics/Appendix2_motor_close.py
      (modified after Joy-it, 2017).
3. Run both Python scripts by executing ‘python motor_open.py’ and ‘python motor_close.py’ to test if the motor is running smoothly in both directions. Pay attention to the motor driver’s red LEDs flashing sequentially to spot possible faulty connections (check the GPIO connections and re-solder joints if single LEDs are dimmer than others).
4. To attach the gate to the motor, we used a 3D-printed spindle disk (PLA-biodegradable 3D printing filament, Figure 1A, Supporting Figure 1B). The 3D printing file is provided at https://github.com/vlucks/Automated_Setup_Gates/tree/main/Appendix2_MotorConnectionandGateMechanics/
   Note that the diameter of the spindle affects the speed at which the gate is opened and closed. Two PCV- U-profiles were used as a rail guiding the movement of the gate (Supporting Figure 1A).


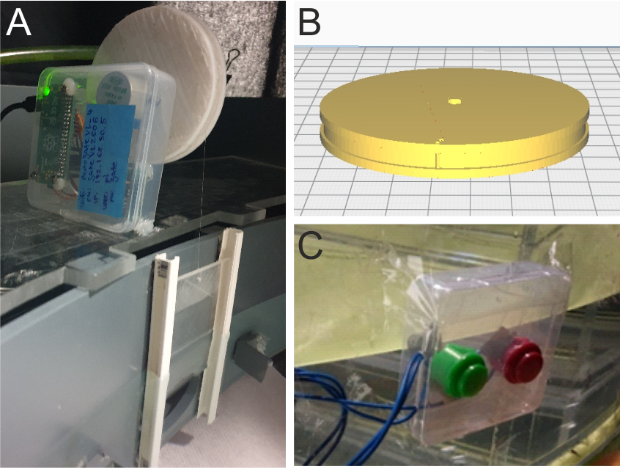


**Supporting Figure 1:** Assembly of the mechanical and electronic components **(A).** The gate is moved upwards by anti-clockwise rotation of the 3D-printed spindle **(B)** that winds up the fishing line connected to the gate **(A)** via the Raspberry Pi’s motor output if triggered by input from pushbuttons **(C)** at the end of the swimming corridor.

**Appendix 3: Motor control by push buttons**

In addition to the parts listed in Appendix 1 & 2 the following parts are required:

1. Two MIYAMA DS412 push-button switches
2. Two 1kΩ metal film resistors
3. Four Copper cables as long as the desired distance between the gate and the placement of the button
4. Soldering iron

Protocol:

1. Work through all steps of Appendix 1 & 2.
2. Solder a 1kΩ metal film resistor to one of each push button’s pins.
3. Connect one of each push button’s pins to the Raspberry Pi’s ground (i.e. pins 34 and 39) using the copper wires while the other cable should be connected to any of the free GPIOs (i.e. 16 and 20; Figure 1B, Supporting Figure 1C).

On the Raspberry Pi’s terminal:

1. Create a new script called ‘main.py’ that should detect button presses to trigger the according motor python scripts. (Note that a ‘bouncing time’ is set to avoid one button press to trigger the script several times due to bouncing voltage; during that time the Pi will not respond to new triggers). Copy the code provided at https://github.com/vlucks/Automated_Setup_Gates/blob/main/Appendix3_MotorControlbyPushbuttons/Appendix3_button_main.py
2. The final step is to make the Raspberry Pi run the main script ‘main.py’ that manages the gate motion as soon as it has booted. This avoids the need to connect to the Pi and starting the script manually each time.
   1. Execute ‘sudo crontab -e’.
   2. At the very end of the file, add the following line:
      @reboot python /home/pi/main.py
3. After rebooting the Pi (‘sudo reboot’), the system should now start automatically. Test if push-button presses move the motor in opposing directions.

**Appendix 4: Remote motor control of setup gates**

In addition to the parts listed in Appendix 1 & 2 the following parts are required:

1. Any digital camera system to observe the experimental animal

Protocol:

1. Work through all steps of Appendix 1 & 2.
2. To control the gates wirelessly, a hotspot is created on the Raspberry Pi (this makes it easily accessible for most platforms). To do so, the protocol RaspberryConnect’s AutoHotSpot (RaspberryPiConnect, 2023) provided at https://github.com/RaspberryConnect/AutoHotspot-Installer/ is used.

On the Raspberry Pi’s terminal:

- 1. Download the Autohotspot package by executing
     ‘curl "https://www.raspberryconnect.com/images/hsinstaller/AutoHotspot-Setup.tar.gz" -o AutoHotspot-Setup.tar.gz’.
  2. Unpack the folder with
     ‘tar -xzvf AutoHotspot-Setup.tar.gz’.
  3. Navigate to the unpacked folder
     ‘cd Autohotspot’.
  4. Run the setup to install the hotspot
     ‘sudo ./autohotspot-setup.sh’.
  5. Select option 2 to install the ‘Autohotspot with no internet for connected devices’.
  6. Select option 7 to customize the hotspot's SSID (i.e. name of created hotspot) and password.
  7. Remember the hotspot’s IP address for ssh access (‘192.168.50.5’ by default).

1. Reboot the system and switch off home wifi; otherwise, the Pi connects to this network and does not open the hotspot. This is done to still have access to the system in case the hotspot fails. (Note that the Autohotspot is started as a service automatically on reboot so there is no need to add it to crontab like in Appendix 3 step 5).

On your PC:

- 1. Wait for a new network to appear in internet-/network-settings.
  2. Sign in to the hotspot of the Raspberry Pi using the defined password (see Appendix 1, step 10).
  3. With any browser, the call of the Pi’s IP address (‘192.168.50.5’ by default) should now show the default webpage of the webserver.
  4. Note that putty connection also works using this IP address once you are connected to the Pi’s hotspot

1. Next, the default webpage of the web server is replaced with a custom webpage that contains virtual buttons to control the gate.

On the Raspberry Pi’s terminal:

- 1. In Raspberry Pi’s OS, navigate to the web server's location
     ‘cd /var/www/html’.
  2. The code for the website is written in PHP. Install the appropriate library with
     ‘sudo apt install php libapache2-mod-php -y’.
  3. “ls” shows the content of the folder – containing an index.html file.
  4. Open the file by typing ‘nano index.html’, change the file ending to ‘index.php’ and copy the PHP code to construct the webpage provided at https://github.com/vlucks/Automated_Setup_Gates/blob/main/Appendix4_RemoteControlofSetupGates/Appendix4_RemoteControlofGates.php
  5. Next, the PHP script needs permission to operate shell commands.
     1. Navigate back to the main directory by ‘cd ..’
     2. Execute ‘sudo visudo’ to modify permissions by adding the following lines to the end of the file:
         #includedir /etc/sudoers.d
         www-data ALL= NOPASSWD:/usr/bin/python3

www-data ALL=NOPASSWD: /usr/sbin/poweroff
 www-data ALL= NOPASSWD:/var/www/html/index.php

- - 1. Adapt access rights further by
        sudo chmod -x /var/www/html/index.php
        sudo chown www-data:www-data /var/www/html/index.php

1. Reboot (see Appendix 1, step 11).

On any wifi-capable device:

1. You can now call the created local website to operate the gate with any wifi-compatible device connected to the local hotspot (e.g. mobile phones, working PC).
   1. Matlab code that shows a live video, acquires a video and calls the created website for motor control is provided at

https://github.com/vlucks/Automated_Setup_Gates/blob/main/Appendix4_RemoteControlofSetupGates/Appendix4_RemoteControlofGates.m

**Appendix 5: Gate triggered by the presence of fish in ROI via Matlab**

In addition to the parts listed in Appendix 1, 2 & 4 the following parts are required:

1. Matlab (suited for connection of any programming software able to call web pages, but shown here in Matlab)

Protocol:

1. Work through all steps of Appendix 1, 2 & 4.
2. Instead of observing the experimental animal via video input in Matlab and opening/closing the gate manually on the Raspberry Pi’s local hotspot (see Appendix 4), the gate should be directly controlled based on the fish’s position (Figure 3A).

In Matlab:

1. For this, two defined regions of interest (ROIs) are continuously checked for the presence of the fish indicated by changes in pixel brightness values.
    ‘min(ROI_1/2(:))<= threshold == 1;’
2. Fish detection in the first ROI triggers the start of video recording and opens the gate (either immediately or after a randomly chosen waiting time). To do so the website is called to open the gate on the Raspberry Pi (i.e. the “virtually clicked” open button).
    ‘web('http://192.168.50.5/index.php?entrance_open=Open+Gate')’
3. When the fish has passed the gate and enters the second ROI, the gate is closed by calling the website to close the gate (i.e. the “virtually clicked” close button). After a short delay, the video acquisition is stopped.

‘web('http://192.168.50.5/index.php?entrance_open=Close+ Gate')’

1. The complete Matlab code is provided at
   https://github.com/vlucks/Automated_Setup_Gates/blob/main/Appendix5_GateTriggeredbyFish/Appendix5_Gate_triggered_by_fish.m

**Appendix 6:** **T-maze: Several gates triggered by the presence of the fish in regions of interest (ROIs) via Matlab**

In addition to the parts listed in Appendix 1, 2, 4 & 5 the following parts are required:

1. Matlab (suited for connection of any programming software able to call web pages, but shown here in Matlab)
2. Three Stepper motors 28BYJ-48 with ULN2003 motor drivers
3. One additional adjustable power supply (here: Voltcraft SNG 12/1500)
4.
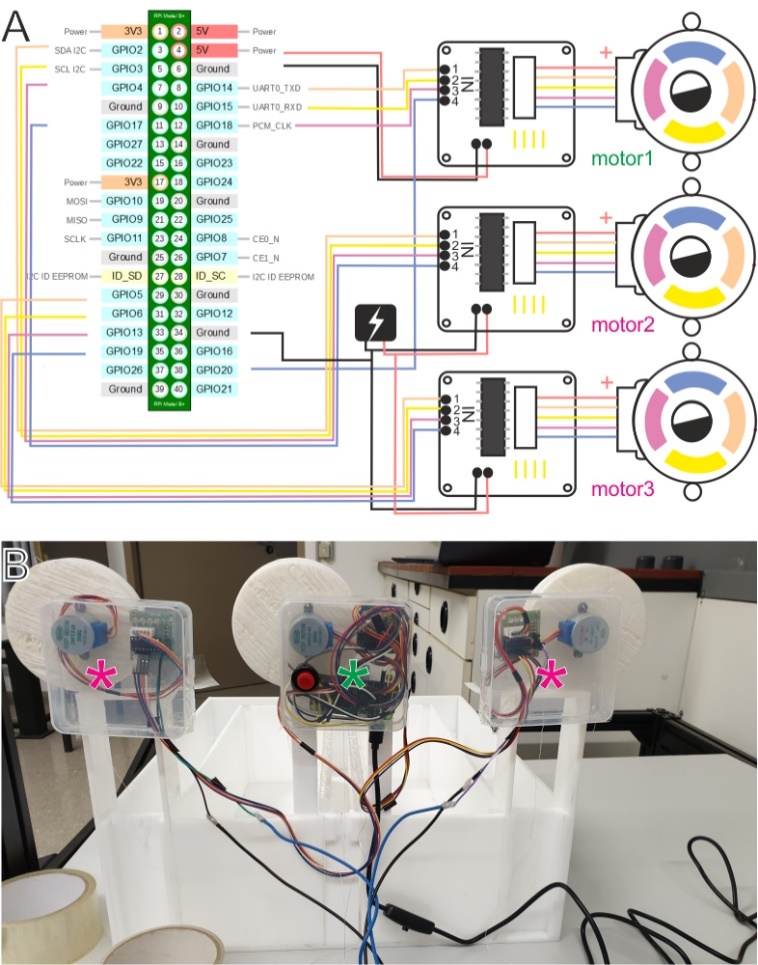
Soldering Iron

**Supporting Figure 2:** Wiring scheme of the automated T-maze with three setup gates **(A).** Indicated GPIOs were used in the exemplary scripts. Note that one motor controller is powered by the Pi and two others by an external power supply. Photograph of the constructed setup **(B)**. The green asterisk indicates entrance gates; the red asterisk indicates decision gates.

Protocol:

A T-Maze with one entrance and two simultaneously moving decision gates (Figure 3B, Supporting Figure 2) is controlled manually via a webpage or automatically in a Matlab script.

1. Work through all steps of Appendix 1, 2 & 4 and 5.
2. Connect the three motors and drivers following the wiring scheme shown in Supporting Figure 2A. Note that the used Pi cannot power three motors. Therefore, one motor is powered by the Pi’s 5V GPIO (see Appendix 2), while the other motors are connected to an external power source (Voltcraft SNG 12/1500).

On the Raspberry Pi’s terminal:

1. Two motor scripts (to open/close the gate) for each motor are created. To control for gate malfunctions and accumulated errors by missed steps (e.g. from power shortages), motor steps and distinct states for opening and closing are monitored. All exemplary files are provided at
   https://github.com/vlucks/Automated_Setup_Gates/tree/main/Appendix6_AutomatedTmaze/
2. Adapt the PHP script to contain additional buttons that run the new motor scripts for the decision gates’ motors 2 & 3 provided at
   https://github.com/vlucks/Automated_Setup_Gates/blob/main/Appendix6_AutomatedTmaze/Appendix6_Tmaze_index.php

In Matlab:

1. In addition to the motor control via the webpage, gates can be controlled automatically based on the fish’s position in ROIs. Two ROIs trigger the opening/closing of the entrance gate and two other ROIs trigger the opening/closing of both decision gates (Figure 3B) in the Matlab example provided at https://github.com/vlucks/Automated_Setup_Gates/blob/main/Appendix6_AutomatedTmaze/Appendix6_Automatic_Tmaze_setup_gates.m

**References**

Lyons, G. (2022). RPiMotorLib. Available at https://github.com/gavinlyonsrepo/RpiMotorLib/blob/master/Documentation/28BYJ.md (last accessed 21 April 2024)

Degger (2014). Arduino stepper. Available at http://makerspace.pbworks.com/w/page/71599862/Arduino%20Stepper (last accessed 21 April 2024)

Joy-It (2017). User Manual Moto1 28BYJ-48 Stepper Motor. Available at https://joy-it.net/files/files/Produkte/SBC-Moto1/SBC-Moto1-Manual.pdf (last accessed 21 April 2024)

Raspberry Pi-StackExchange (2019). GPIO Pinout Orientation Raspberry Pi Zero W- Available at https://raspberrypi.stackexchange.com/questions/83610/gpio-pinout-orientation-raspberypi-zero-w (last accessed 21 April 2024)

RaspberryPiConnect (2023). AutoHotspot-Installer. Available at https://github.com/RaspberryConnect/AutoHotspot-Installer (last accessed 22 April 2024)
